# A hypothesis on chemical mechanism of the effect of hydrogen

**DOI:** 10.1186/2045-9912-2-17

**Published:** 2012-06-21

**Authors:** Penghui Shi, Wancang Sun, Pengzhong Shi

**Affiliations:** 1 Gansu Key Lab of Crop Improvement & Germplasm Enhancement, Gansu Provincial Key Laboratory of Aridland Crop Sciences, Gansu Agricultural University, Lanzhou 730070, China; 2 Inner Mongolia University of Science and Technology, School of Material and Metallurgy, Baotou 014010, China; 3 Gansu Agricultural University, Anning District, Lanzhou City, Gansu Province, China

**Keywords:** Hypothesis, Hydrogen, Mechanism, Ligand

## Abstract

Many studies have shown that hydrogen can play important roles on the antioxidant, anti-inflammatory and other protective effects. Ohsawa et al have proved that hydrogen can electively and directly scavenge hydroxyl radical. But this mechanism cannot explain more new experimental results. In this article, the hypothesis, which is inspired by H_2_ could bind to the metal as a ligand, come up to explain its extensive biology effect: Hydrogen could regulate particular metalloproteins by bonding (M–H_2_ interaction) it. And then it could affect the metabolization of ROS and signal transduction. Metalloproteins may be ones of the target molecules of H_2_ action. Metal ions may be appropriate role sites for H_2_ molecules. The hypothesis pointed out a new direction to clarify its mechanisms.

## Background

**Figure 1:**
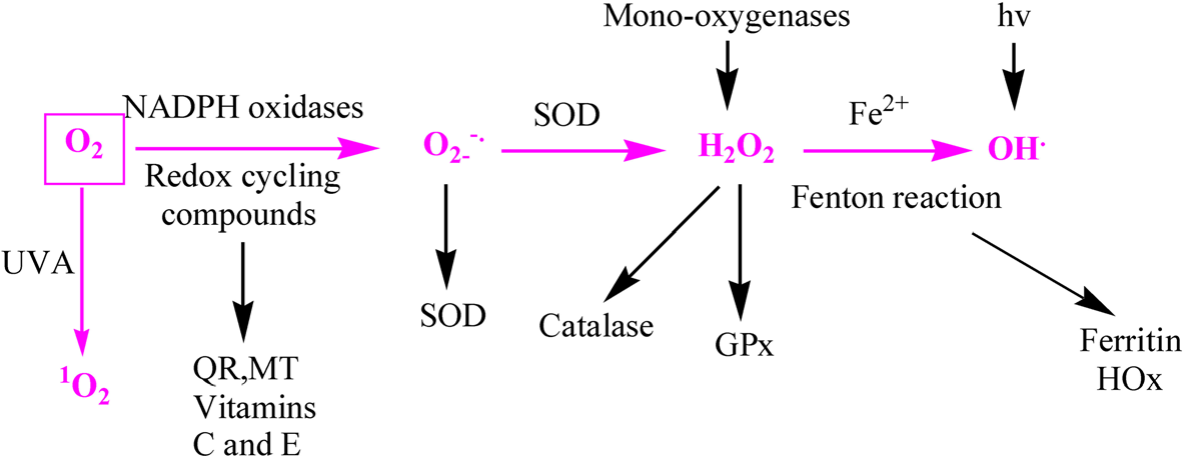
**ROS generation and detoxification. **Various chemical reactions, with or without enzymic catalysis, generate ROS. The dioxygen molecule undergoes successive reductions which yield the superoxide radical anion (O_2−_), hydrogen peroxide (H_2_O_2_) and the hydroxyl radical (OH·). Antioxidant systems act as ROS scavengers to maintain the intracellular redox status. Quinone reductase (QR) detoxifies quinone compounds, metallothionein (MT) traps (heavy) metal cations, and vitamins C and E trap free radicals. SOD and catalase respectively dismutate superoxide (into oxygen and hydrogen peroxide) and hydrogen peroxide (into oxygen and water). Glutathione peroxidase (GPx) acts like catalase on various peroxide compounds, including H_2_O_2_[5].

Basal cellular metabolism continuously produces reactive oxygen species (ROS). O_2_ may generate successively superoxide (O_2−_), hydrogen peroxide (H_2_O_2_) and hydroxyl radical (OH^·^). ROS are able to oxidize biological macromolecules such as DNA, protein and lipids [1,2]. Some enzymic systems detoxify ROS, however, catalase dismutates H_2_O_2_, and SOD eliminates O_2−_ (but generates H_2_O_2_). Excess oxygen can react with H_2_O_2_ to produce hydroxyl radicals by the Fenton reaction [3] (Figure 1). Ohsawa et al provide evidence that hydrogen could reach subcellular compartments such as the nucleus and mitochondria, biochemical experiments using fluorescent probes and electron paramagnetic resonance spectroscopy spin traps indicated that hydrogen gas may selectively scavenge the hydroxyl radical [4]; They were the first to show the ability of H_2_ to suppress oxidation in vivo. So far, many researches have proved the central role of hydrogen on the antioxidant, anti-inflammatory and other protective effects.

Much evidence has shown that hydrogen exert beneficial effects in animal models of a number of diseases mainly associated with oxidative stress; However, the findings don’t cover plants. Moreover, the mechanism of the effect of hydrogen remains unclear, the most acceptable mechanism is that the hydrogen can electively and directly scavenge hydroxyl radical while preserving other reactive oxygen and nitrogen species important in signaling [4]. There is some question, however, the published rate constant for the reaction of ^·^OH with H_2_ to form H_2_O and H^·^ is drastically slower than most radical-radical reactions [6]. Furthermore, it can’t explain some new experimental results. For example, Tomohiro Itoh proved that hydrogen exerts its beneficial effect by modulating some signaling pathways. Experimenters found that oral intake of hydrogen-rich water abolishes an immediate-type allergic reaction in mice. The results indicated that hydrogen attenuates phosphorylation of the FcεRI-associated Lyn and its downstream signal transduction, which subsequently inhibits the NADPH oxidase activity and reduces the generation of hydrogen peroxide. they also found that inhibition of NADPH oxidase attenuates phosphorylation of Lyn in mast cells, indicating the presence of a feed-forward loop that potentiates the allergic responses. Hydrogen accordingly inhibits all tested signaling molecule(s) in the loop. The results imply that effects of hydrogen in some diseases are possibly mediated by modulation of yet unidentified signaling pathways [7].

### Hypothesis and discussion

Although the beneficial effect of hydrogen is generally accepted, the mechanism is not still clear. There can be little doubt that biology function of H_2_ depends on the physical and chemical interaction of other molecules with it , so what would it be like?

**Scheme 1:**
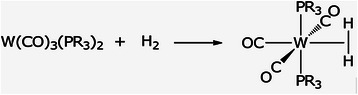
Formation of a dihydrogen complex.

In organometallic and inorganic chemistry, for some metal complexes, the "arrested" addition product can be isolated–the dihydrogen complex is obtained as a stable species that can be put in a bottle: [8] (Scheme 1).

**Figure 2:**
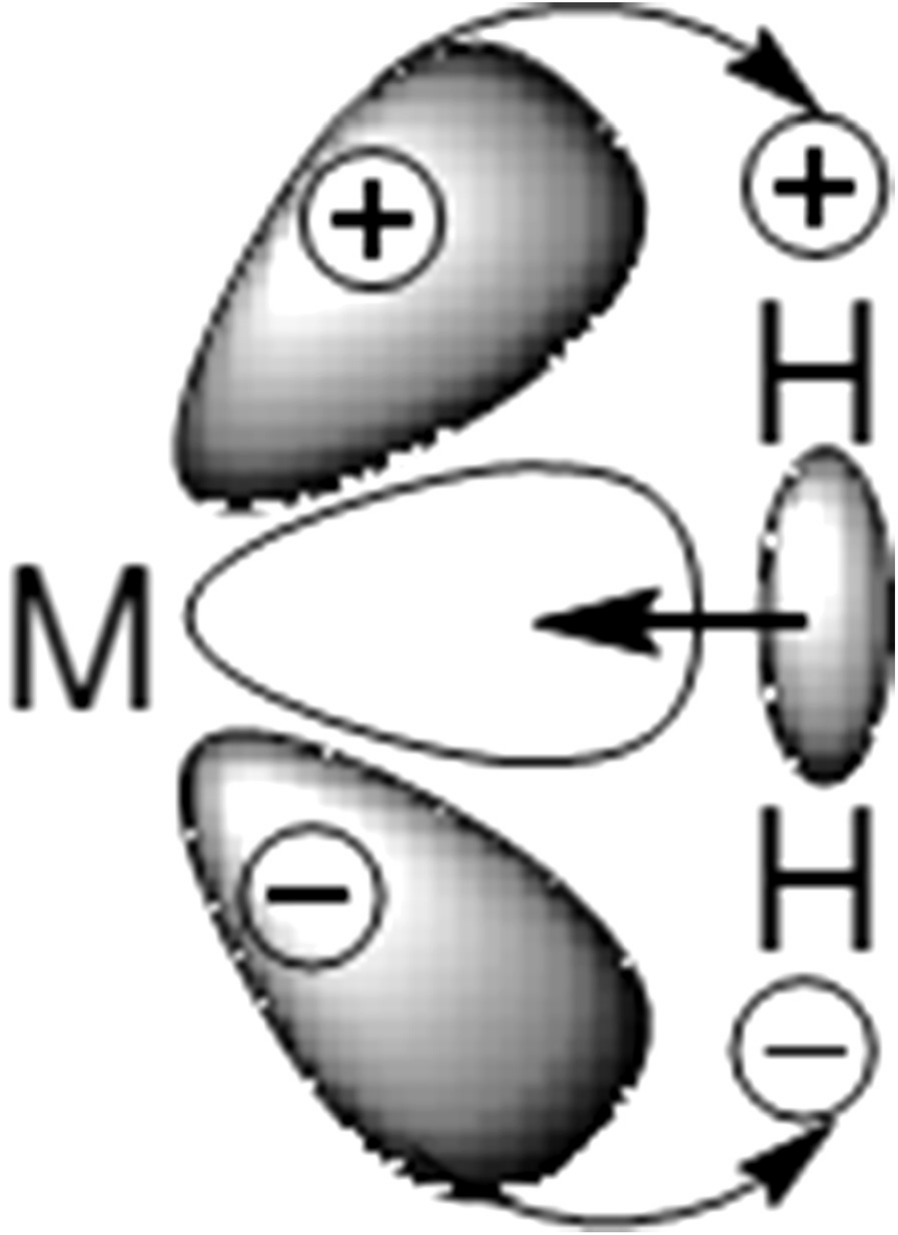
**Schematic bonding model between molecular hydrogen and a metal **[14]**.**

**Figure 3:**
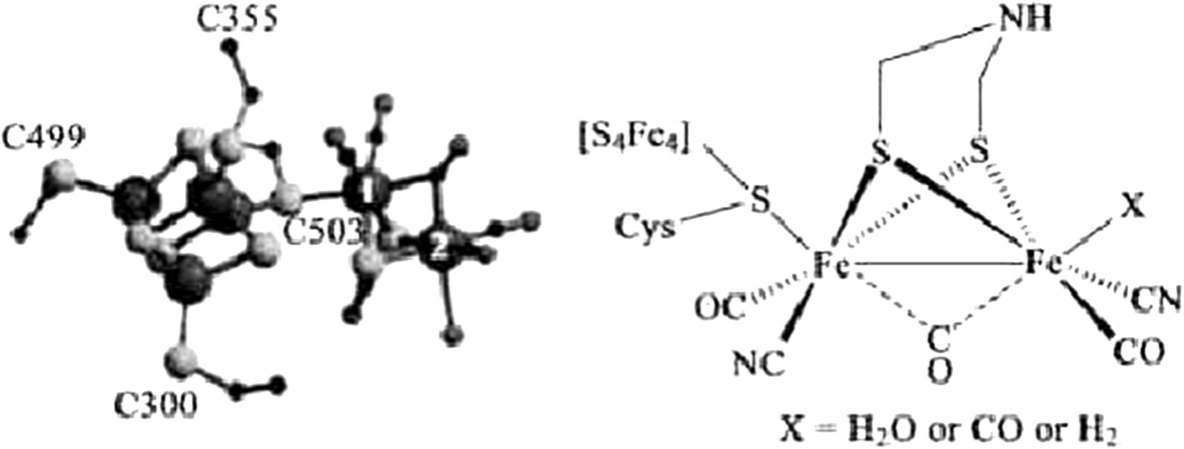
**Stereo view of the CpI H-cluster and coordinating Cys ligands located at the boundary of the two lobes of the active site domain **[15].

Traditional ligands use lone pairs of electrons in their bonding, but in dihydrogen complexes, the bonding to the metal comes from donation of electron density from the nonpolar H-H σ bond to d orbitals on the metal (Figure 2). We hold that hydrogenase is a typical case in point. Hydrogenases catalyse the reversible oxidation of molecular hydrogen (H_2_). The active site domain of the Fe hydrogenases contains an unusual Fe-S centre termed the H-cluster. H-cluster consists of the [Fe_4_S_4_ subcluster bridged via the Cys thiolate to the [Fe_2_ (binuclear iron) subcluster (Figure 3) [9-12]. Iron sulfur clusters are found at the active sites of numerous enzymes where they commonly facilitate electron transfer and substrate transformations (Table 1) [13]. It is infered that M–H_2_ interaction also exists in these metalloproteins. By extension, it also should exist in non-cluster metalloproteins. All this suggestes that metal ions may be the site where H_2_ interacted with metalloproteins.

**Table 1:** Iron-sulphur proteins in cells

**Function**	**Protein class**
Catalysis	Bacterial nitrate reductase, Formate dehydrogenase, Fumarate reductase, Glutamine PRPP amidotransferase, Hydrogenase, Methane monooxygenase, NADH:ubiquinone reductase, Phthalate dioxygenase reductase, Succinate dehydrogenase, Sulphite reductase, Xanthine dehydrogenase, Aconitase (TCA cycle)
Electron transfer	Ferredoxins, Rieske proteins, Rubredoxins, NADH Dehydrogenase, Succinate-CoQ Reductase, CoQ-cyt c Reductase (respiratory chain complexes)

It is proved from experiments that molecules containing a metallic cation may promote O_2−_ formation because they have the ability to store and easily give an electron to molecular dioxygen [16]. Free radicals arise through the autoxidation catalyzed by metalloproteins, this mainly occurs within the mitochondria. Some experimental results proved that hydrogen can permeate into mitochondria and prevent superoxide formation [17]. It indicates that H_2_ can affect the metabolization of ROS by way of superoxie formation. And it must have a big impact on the content of H_2_O_2._ Besides, free radical production can also happen in other cellular compartments, such as NADPH oxidase. ROS production can interfere with signal transduction pathways [18-20]. ROS, in particular H_2_O_2_, are indeed second messengers for many physiological stimuli, some stimuli have been proven to induce mitochondrial H_2_O_2_ release [21].

On the basis of the mentioned analysis, we evaluate that metal ions could be appropriate role sites for H_2_ molecules. We propose that hydrogen can permeate into mitochondria and concentrate at mitochondrial membrane to regulate activity of metalloproteins (complexesI,II,III) by M–H_2_ interaction. The same may be true of NADPH oxidase. This could reduce the production of superoxide and prevent synthesis of the hydroxyl from the source. Hydrogen, on the one hand, maybe regulate the content of H_2_O_2_ by affecting the metabolization of ROS. And then it disturbs signaling pathway. On the other hand, hydrogen maybe directly influence signal transduction by regulating particular metalloproteins of signaling pathways. It may be the mechanism of its extensive biology effect.

## Conclusions

We propose that metalloproteins may be ones of the target molecules of H_2_ action. Metal ions may be appropriate role sites for H_2_ molecules. And in this way can we explain its extensive biology effect. Although some details remain murky, the hypothesis pointed out a new direction for the continuation. It is a good inspiration to clarify the mechanism of the effect of hydrogen. We predict that hydrogen can affect many metalloproteins activities.Therefore, more studies will be necessary to test the hypothesis.

## References

[B1] Vaughan M Oxidative modification of macromolecules minireview series J Biol Chem 1997 272 18513 10.1074/jbc.272.30.18513

[B2] Hagen TM Ingersoll RT Lykkesfeldt J Liu J Wehr CM Vinarsky V Bartholomew JC Ames AB (R)-alpha-lipoic acid-supplemented old rats have improved mitochondrial function, decreased oxidative damage, and increased metabolic rate FASEB J 1999 13 411 418 9973329 10.1096/fasebj.13.2.411

[B3] Woo ES Lazo JS Nucleocytoplasmic functionality of metallothionein Cancer Res 1997 57 4236 4241 9331083

[B4] Ohsawa I Ishikawa M Takahashi K Hydrogen acts as a therapeutic antioxidant by selectively reducing cytotoxic oxygen radicals Nat Med 2007 13 688 694 10.1038/nm1577 17486089

[B5] Morel Y Barouki R Repression of gene expression by oxidative stress Biochem J 1999 342 481 496 10.1042/0264-6021:3420481 10477257 PMC1220487

[B6] Wood KC Gladwin MT The hydrogen highway to reperfusion therapy Nat Med 2007 13 673 674 10.1038/nm0607-673 17554332

[B7] Itoh T Fujita Y Ito M Molecular hydrogen suppresses FcRI-mediated signal transduction and prevents degranulation of mast cells. Biochem Biophys Res Commun 2009 389 651 656 10.1016/j.bbrc.2009.09.047 19766097

[B8] Kubas GJ Ryan RR Swanson BI Vergamini PJ Wasserman HJ J Am Chem Soc 1984 106 451 452 10.1021/ja00314a049

[B9] Darensbourg MY Lyon EJ Smee JJ The bio-organometallic chemistry of active site iron in hydrogenases Coord Chem Rev 2000 219/221 533 561

[B10] Marr AC Spencer DJE Schurder M Structural mimics for the active site of [NiFe] hydrogenase Coord Chem Rev 2001 219/221 1055 1074

[B11] Nicolet Y de Lacey AL Vernde X Crystallographic and FTIR Spectroscopic Evidence of Changes in Fe Coordination Upon Reduction of the Active Site of the Fe-Only Hydrogenase from Desulfovibrio desulfuricans J Am Chem Soc 2001 123 1596 1601 10.1021/ja0020963 11456758

[B12] Lawrence JD Li HX Rauchfuss TB Angew Chem Int Ed 2001 40 1768 1771 10.1002/1521-3773(20010504)40:9<1768::AID-ANIE17680>3.0.CO;2-E 11353506

[B13] Cowan JA Inorganic Biochemistry: An Introduction 1993 New York: VCH Publishers

[B14] Martin HG Prechtl 2007 WiKu: Publisher

[B15] He Chengjiang WangMei Li Minna Sun Licheng Advance in chemical mimic of fe-only hydrogenase Progress in chemistry 2004 16 2 250 255

[B16] Kagan VE Tyurina YY Recycling and redox cycling of phenolic antioxidants Ann N Y Acad Sci 1998 854 425 434 10.1111/j.1749-6632.1998.tb09921.x 9928449

[B17] Sato Y Kajiyama S Amano A Hydrogen-rich pure water prevents superoxide formation in brain slices of vitamin C-depleted SMP30/GNL knockout mice Biochem Biophys Res Commun 2008 375 346 350 10.1016/j.bbrc.2008.08.020 18706888

[B18] Finkel T Oxygen radicals and signaling Curr Opin Cell Biol 1998 10 248 253 10.1016/S0955-0674(98)80147-6 9561849

[B19] Lander HM An essential role for free radicals and derived species in signal transduction FASEB J 1997 11 118 124 9039953

[B20] Suzuki YJ Forman HJ Sevanian A Oxidants as stimulators of signal transduction Free Radical Biol. Med 1997 22 269 285 10.1016/S0891-5849(96)00275-4 8958153

[B21] Quillet-Mary A Jaffrezou JP Mansat V Bordier C Naval J Laurent G Implication of mitochondrial hydrogen peroxide generation in ceramide-induced apoptosis J Biol Chem 1997 272 21388 21395 10.1074/jbc.272.34.21388 9261153

